# A Letter to Doctor Joseph Adams, on the Hydatid Existence of Cancer

**Published:** 1806-05

**Authors:** Samuel Young

**Affiliations:** No. 22, Welbeck Street


### Medical and Physical Journal.

VOL. XV.] MAY, 1806. [NO. 87.

*Printed for R. PHILLIPS, by W. Thorne, Red Lion Court, Fleet Street, London.*

### *To the Editors of the Medical and Physical Journal.*

GENTLEMEN,

IT probably is as well to anticipate the surprise that naturally would attend the publication of a Letter, in Answer to an Hypothesis made known at least four years ago, and which might have been so easily noticed in a late publication, (An Inquiry into the Nature and Action of Cancer, by the Author, last July). The fact is, strange as it may appear, the Author never knew such an hypothesis was in being till some time after the publication of his Inquiry, although he had made no inconsiderable search after works on Cancer. It is indeed matter of great surprise, that an Hypothesis of such notoriety, coming from an author of such celebrity as Dr. Joseph Adams, and addressed to the first physiologists of the age, should neither have drawn an attempt at refutation on the one hand, nor claimed the public devoirs of proselytes on the other. A baneful indifference too long has enveloped the subject. The Author is ashamed that an appearance of neglect should have attached to him, and hastens to make all the reparation in his power.

*March* 5, 1806.

### A LETTER TO DOCTOR JOSEPH ADAMS, ON THE HYDATID EXISTENCE OF CANCER.

SIR,

SINCE every hypothesis can only be respectable, as far as it is supported by general experience and facts, I am confident you will not be inimical to an inquiry so governed, touching your opinion of the hydatid existence of cancer.

cancer.* And since the importance of the subject (upon which you have displayed so much classical ingenuity,) must be more or less interesting to every one, a freedom of investigation is allowed, and which, in such an instance, may be fully enjoyed, without the slightest dereliction of personal respect. I am confident your researches have aimed to establish the real nature of the disease, and not merely to build up a splendid hypothesis; under this impression, therefore, I shall not hesitate without further preface, entering upon the merits of your conclusions, which I am confident you would have every one do, before he adopted them.

I frankly confess, Sir, that however I may admire the novelty and ability of your hypothesis, considerable discordancy would appear to arise in its application: it would seem not altogether to be drawn *simply* in regular progression from what *really is:* but on the contrary, a *solitary appearance* (the contraction of the fatty cells, which is in no way constant, because the diseased structure, in which such cells are contained, is not always present in scirrhus) has given rise *(apparently)* to a false presumption in the first instance, which is attended by a train of ingeniously framed observations, as its consequences and support.

In way of prelude to the hydatid existence of cancer, you have prefixed, "An account of the dissection of a man that died of a suppression of urine, produced by a collection of hydatids between the neck of the bladder and rectum, by Dr. John Hunter, F. R. S. &c." wherein is established the separate animal existence of the hydatis lymphatica. You then proceed, in a letter to Dr. Baillie, to explain the simplicity of the life of the hydatid, by transcribing a part of "The Morbid Anatomy." "Life may be attached to the most simple form of organization. In proof of this, hydatids have been found in the brains of sheep, exactly resembling those of the human liver, and which have been seen to move; and therefore are certainly known to be animalcules: The hydatids, indeed, of the human liver have not, as far as I know, been found to move, when taken out of the body and put into warm water; were this to have happened, no difficulty would remain."

* Vide, The Doctor's Observations on the Cancerous Breast, consisting chiefly of original correspondence between the Author, and Dr. Baillie, Mr. Cline, Dr. Babington, Mr. Abernethy, Dr. Stokes, &c.

main." (Vide Morbid Anatomy, 2d edition, p. 225.) And upon a parity of reasoning which you draw from the contractile power of the coats of the hydatis lymphatica, you endeavour to rest the separate existence of the cells, often observed in the fat of cancerous structures. "No more (you say) will be required of you, than to prove the same properties (alluding to the contractile power of the hydatid coats) unconnected with elasticity in the fatty part of the cancerous breast." The demonstration of which, you reduce in the following manner.

"Immediately after the operation, take the amputated part, and cut it in a transverse, or indeed, in any direction, and wherever you discover this fatty appearance, you will see the surface at first smooth under your knife; in an instant after, you will find a papillary appearance all over the yellow green surface. Each of these papillæ you will find to be part of the contents of a capsule, the contraction of which has produced this conical figure. If the amputated part has been exposed long to the cold, or thrown into water, and left there for several hours, a section of it no longer exhibits this appearance, nor is it possible, in this stage, to distinguish at first sight, what I call carcinomatous hydatids from common fat. By this, it appears, that the yellow green transparency of the fat, and the contractile power of the enclosing tunics, could only arise from life, and the degree of heat with which that life was attended."

Now, even admitting that the contractile power of these cells is clearly demonstrated by this, their *separate animal* existence is by no means made out to authorize the classical term you have given of *hydatis carcinomatosa.* I must beg leave here to remark, Sir, that your hypothesis falls into the *separate existence* of these cells so instantaneously, that one is compleatly taken by surprise; and the only resource left one, is firmly to persist in calling out for proof. Here, we certainly want that part of Dr. Baillie's letter answered, where he hints at the *necessity* of a just and absolute distinction, between a *separate animal existence* and the *common life of* a part.

"Respecting the propriety of the term cancerous hydatid, (replies Dr. Baillie) I have only to remark, that if you can prove the cyst in a schirrous structure to possess a life peculiar or proper to themselves, like the hydatids found in the liver, there would seem to be no impropriety in the term. But if they possess merely a living principle, similar to that of the ordinary diseased structures in an animal body, I should think it better for you to adhere to the name of cyst."* On this point therefore, Sir, I must beg to remain at issue, till the *separate animal existence* is better established in cancerous structure, than merely by the solitary "papillary appearance" you have noticed.

In supposing these cells to be hydatids, we are obliged to commit a violence to the evidence of our senses; because we cannot forget that the breast of a woman is a *bed* of fat, and therefore we are by no means surprised to find small cells in the cancerous breast containing diseased fat; it is what we naturally look for: nor is the alteration by any means more than what we rationally can account for: no stretch of faculty is required to answer the obliteration of the lateral communications: Do we not see it every day in common inflammation? The sides of the cells are thickened. Do we not see this in every instance of abscess? I cannot therefore imagine why we should lose sight of original structure, or of known simple diseased actions, when we go into an inquiry of more complicated alterations; and why, instead of thus progressively following up disease, we should entirely wash out all remembrance of natural actions and original structures, and set about a new creation. In the altered membranous structure in cancer, (when it is present) where is there the marked characteristic of independent existence, with regard to the connecting cellular and adipose membrane? If the morbid change has not extended to the whole membranous structure of the breast, cannot one trace the progress of the alteration from the *diseased fat* in *intimate connexion* to where the line of difference is imperceptibly lost in *healthy structure*? Or, vice versa, cannot one commence with the *healthy structure*, and by progressive connexion

* The evidence necessary for a separate existence would appear not altogether to be sufficiently marked by the distinction as it here stands: Common life, and "proper life." Strictly speaking, there cannot be *two* distinct sorts of life. The different *degrees* of life, no doubt, are endless. Variously modified, and combined, it may be; yet, however, *life* can only be *life* after all. The common life of a part is its "proper life," and therefore unquestionably "peculiar" to that part. The life of a bone is as peculiar and proper to it, as *that* of the most irritable muscle: The *degree* only of the self-same principle constituting the *difference*. To prove therefore, a separate animal existence in these cells, it is not sufficient to say, that they possess a higher degree of life, than what is commonly given to morbid structures. The evidence necessary for such a conclusion is, that they should have a *distinct* chain of functions, evidently arising out of a *separate* and *peculiar œconomy* of their own.

nexion be led into the *morbid alteration?* In fact, is there not such an intimate blending and similitude between the two structures, tha the line of difference between the healthy and the diseased is perfectly indefinite? or, is there that difference between the *two*, that any one can with ease immediately point out the distinction,. and say, this is the morbid structure; — here begins the hydatid mass; — this could never have been *simple adipose?* No, on the contrary, the whole weight of the distinction hangs upon a hair. This structure, we are told, cannot be distinguished in appearance (some time after its amputation) from *common fat*; and it is only by minute examination, and by cautiously picking out of the fat, that we find it less intersected than *healthy* adipose, and that the cells in which it is contained are more distinct, and their sides more dense than those of common cellular structure. Upon *this difference* rests the proof of the animalcular existence in a carcinomatous breast. And all that can be drawn in support of a *separate animal œconomy*, is the *indistinct* evidence of a *questionable* fact; viz. " the papillary appearance in recently amputated cancerous fat." Touching, therefore, the independent existence of the cells in cancerous fat, (though vessels may be obliterated, and many changes wrought which never existed in health) if such be admitted, then, in my mind, every adipose cell in the body is likewise an hydatid.

From the particular notice you take of the green transparency of the cancerous fat, observable just after amputation, (and which it loses if long exposed to cold, or if it is soaked many hours in water, when it can no longer be distinguished from common fat) you undoubtedly, Sir, seem to attach a peculiarity exclusively derived from the circumstances attendant on the life of the part. For, speaking of this appearance on the one hand, and the contractile power of the fatty tunics on the other, you say, they " could only arise from life, and the degree of heat with which that life was attended." Such an observation could only be made with the view of attaching a peculiarity — of proving some exclusive property to the part: without some such intention, the conclusion would never have been made. I cannot see that the green transparency of such fat has any thing to do with the life of the part, or any property arising out of it as a *necessary principle*. The *common dégree* of heat attendant on life is fully adequate to render any fatty substance less compact, (whether in or out of the body) than " when it has been

 long long exposed to cold;" and we know fats, when in a liquid state, have a greenish cast: there cannot, therefore, be any extraordinary inference drawn from such an appearance in a mass of fat which has long been exposed to disease, and where so many foreign aids might concur in giving it that appearance. Such, for example, as a diffusion of serum in the cells from some past increased action, and which had not afterwards been wholly taken up.

To account for the alteration in the appearance of this diseased fat after any long exposure either to air or water, we must first of all consider, that the *common* deprivation of heat may contribute in a great measure towards such a change. Or, the coloring matter of such diseased fat, by exposure, or by being suffered to remain many hours in water, might either be washed out on the one hand, or bleached by the action of oxygen on the other. Such changes are daily effected on variously discolored fatty and waxy substances, and where the agency of life is out of the question. However, Sir, supposing "the yellow green transparency," in recently amputated cancerous fat, wholly to rest even on the life of the part, or, directly upon the heat arising from such life, no peculiarity is proved by it: nor can the conical figure you have discovered on the surface of a section of such fat *(alone)* attach a peculiarity to the part.* Here are appearances, and they depend upon the life of the part. What then? No one I believe ever doubted, or will doubt, but what cancerous structures possess a certain degree of *life*, in common with other parts. So that admitting these appearances to originate in the fullest sense of your meaning, *particularly* and *exclusively* from "life, and the degree of heat attendant on that life," such evidence only goes to prove, what was never doubted, viz. A living principle in cancerous structures.

I must beg your patience in returning to the "papillary appearance" you have discovered in cancerous fat, and which you attribute to the contraction of its several tunics, as that is the principal and indeed only point upon which you can rest the separate or hydatid existence of the disease. The conclusion you draw, however, is not founded *directly* on the fact itself, but on *inference* only; and then the rest of the hypothesis is made up by extracts from the life and death of the common hydatid, (which, by the bye, you take for granted is the same in carcinoma ingeniously adapted to the several appearances afforded by the

* "A peculiarity' of course is here used, in allusion to an animalcular œconomy.

the progress of cancer.* You say, the contraction of the hydatis lymphatica proves its separate existence; and then you add, " if I prove this same power in the cells of cancerous fat, I then establish the same thing, i. e. the separate existence of these cells." But permit me to ask, is contraction of their coats alone, the only proof of the separate existence of hydatids?† Would this have been sufficient to maintain their separate animal existence, supposing (instead of being found wholly detached and floating in a fluid) they had been in intimate connection with the surrounding parts, and blended with the common connecting membrane. What gave the idea of separate animal existence to hydatids in the first instance? Not the contraction of their coats; *that* was the confirmation only. It was the perfect independency of these bodies from the surrounding parts; found, as they were, floating in fluid, in which they had been generated, and in which they had multiplied, which first suggested the idea of an independent animal economy of their own: the contraction of their coats was only the common evidence of their life, which subsequent evidence, combining with these primary circumstances, placed their separate existence beyond dispute. In short, Sir, *contraction* of a part alone, is by no means adequate to prove a *separate* existence; and I appeal to yourself, whether the cells of cancerous fat are in any way proved to be unconnected, or otherwise independent, in exception from the surrounding parts, of the common circulating medium. I am free, therefore, to confess, that what you term carcinomatous hydatids, appear to me nothing more than common adipose cells, altered by disease; and even admitting that they possess a property of contraction, which they never possessed in a healthy state; I have only to go one short step farther, and say, that they have acquired that from disease, which they never possessed in health.

Permit me, Sir, to refer you to the fifth letter in your publication, which is from Mr. Cline. It is in allusion to one (I suppose) you addressed to that gentleman, but

 which

* In the hydatis lymphatica, the evidence of a regular growth and decay is afforded: From the minuteness of the smallest pin's head to a diameter of an inch and a half, and so on, these bodies can be distinctly traced, till they waste to an isinglass substance: In carcinoma, all this is left to be imagined.

† Since the contractile power of fibres rests upon the most simple possession of life; the principle of irritability; no peculiarity can possibly attach to the contraction of a part alone.

which does not appear in your publication. I refer you, Sir, to this letter, because it would appear, that you had been making some intimate inquiries about encysted tumors in way of support to your opinions on cancer. Mr. Cline informs you, "that he had lately taken out a small tumor from the breast, which was without the smallest appearance of cavity," but that he "has also met with cavities in such tumors, containing a greenish yellow gelatinous substance in distinct cells;" and that in other instances similar cells have contained "different fluids, sometimes like serum, and in others of a dark bloody appearance." But so far as he had hitherto observed, they did not give the idea of being living hydatids; that is, (as he emphatically remarks) a perfectly circumscribed membrane, without any communicating vessels from the surrounding parts." This is the absolute characteristic of hydatid existence: the *body must be perfectly circumscribed* and *detached.* Therefore the analogy you draw, Sir, in letter vi. however ingeniously applied, certainly does not bear you out. "It has never yet been disputed (you say) that hydatids may be filled with a bloody fluid, as well as with lymph: the case communicated by Watson, in the Philosophical Transactions, was a cluster of hydatids, some of which were filled with lymph, and others with a bloody fluid. They were attached to a "spongy substance, answering the purpose of a placenta." Now, if these were hydatids, I cannot see in what respect they differ from those cysts, which are often found in cancerous breasts, filled with the same fluids, and attached to a spongy substance, which I call the fungus."*

Now, Sir, this cluster of bags was called hydatids, not from their *own independent existence,* but from an assumption of evidence from their strong resemblance to bodies which have been found perfectly detached, and independent of any connecting medium with the surrounding parts. "Hydatids are not a part of the animal in which they are found,

* Some degree of irregularity would seem to exist here. We are led from *small cells,* containing diseased fat, to *cysts,* which are often found in cancerous breasts, containing variously coloured fluids. Such cysts are out of the question, entirely from our present purpose. In an *indirect point* of view, no one can doubt an hydatid existence in a cancerous breast, as a *chance production.* But *now,* we are talking of the little cells, or tunics, observable in cancerous fat (and which, by the bye, are so indistinctly marked, as scarcely to be known from *common fat*) as the *direct origin,* or rather, primum mobile, of the disease.

found, any more than the worms in the intestinal canal."* So that in fact such attached bags, as noticed by Watson, can only take the rank of hydatids, as it were, from courtesy. They depended upon "a spongy substance," from whence they drew their supply, and by which they were connected to the surrounding parts, so that (in this point of view) a separate animal existence in them must be out of the question. From such formations only, hydatid existence (as it is now understood), would never have been thought of, and therefore so far from their establishing the separate existence of other attached cysts, they cannot confirm their own.

In page 52, Mr. Cline informs you, the tumor he removed from the back, and which, he supposes, is the one that you allude to, weighed sixteen pounds, and was entirely adipose; you are also informed, Sir, that *the vessels* were secured by ligatures, as they were divided. Although, in your Answer, no notice is taken of this circumstance, I should suppose the object of your inquiry to have been the *blood vessels,* since you have extended the *hydatid existence* to all *encysted tumors.* If this were the object of your inquiry (and, indeed, I see no other connected with the question) may I ask, Sir, for a further explanation as to the hydatid existence of these tumors? I ask it in the way of instruction, because I am much more inclined to doubt the facility of my own comprehension than your perspicuity. However, to me, Sir, there is an evident difficulty in this part of your hypothesis. At one time the distribution of blood-vessels on their surfaces is mentioned (page 59); and at another time, their independency from the surrounding parts (page 74). Are we to consider the containing cysts of such tumors hydatids? or, are we to attribute such a nature only to their contents? That blood-vessels do not shoot into the contents of steatoma, and the rest of encysted tumors, is surely no evidence whatever to support "a separate animal existence," although they may increase and diminish. Such diseased depositions have regulating cysts upon which their increase and diminution depend; and which, in their turn, derive their blood-vessels in common from the connecting medium.† So that viewing *one* as a diseased action,

---

* Vide Mr. Cline's letter.

† If we refer to less complicated morbid changes, the formation of these cysts would appear wholly and consistently to rest upon a principle of nature; action, and the *other* as in a state of health, I see no specific difference between a *cell* of adipose membrane, and one of these *cysts*, containing a quantity of morbid fat. In the instance of the adipose cell, we see its contents often *diminished*, and as frequently *increased;* no *blood-vessels* can be traced into such deposition, yet (to use the hydatid phraseology) such "growth" and "decay," were never attributed to any *peculiar property* of *separate life*. If, therefore, we see an adipose cell rapidly increasing its contents, or, at times, as quickly diminishing them, and this without any peculiar properties of *separate life*, attributable either to the *cell* or to its *contents*, I am at a loss to conjecture, why such should not be allowed (precisely the same) to the "cyst," or to the "contents" in steatoma."*

You have drawn, Sir, a comparison between such cysts (when their contents have been discharged, as in meliceris) and natural cavities; such, for example, as the tunica vaginalis

---

nature; the shutting out of disease as much as possible from the constitution at large: upon the same rule that the cellular membrane becomes obliterated in its lateral communications, and condensed to serve the purpose of a cyst in common abscess. The different arrangement of the blood-vessels on the surfaces of these cysts, compared to the manner in which vessels are often shortened, and plugged up in the instance of common abscess, cannot surely afford a sufficiency of evidence for the question. A difference in the arrangement of vessels unquestionably is proved; but is it fair to conclude upon this difference, that the whole is an arrangement for a separate and distinct economy? The *one* is a *positive* provision for the while, answering the necessities, and anticipating the accidents of a *temporary* derangement. The *other* is an *unequal*, though systematic effort, to guard against slow and progressive innovation, from the *permanent subversion* of healthy arrangement. The difference in the distribution of the vessels on the cyst therefore, I humbly submit to rest solely and wholly on the necessities of the case, compared to that of common abscess. In the former, no shortening or plugging up of vessels is necessary, because there is no bursting of integuments, nor discharge of contents intended, and therefore no hazard of hæmorrhagy is risked. In the instance of the abscess, Nature sees the evil will redress itself. She says, "all that I have got to do, is to guard against accidents; nothing more is necessary than to wait quietly three or four weeks, and then I shall get rid of the burthen altogether." But in the instance of permanent derangement no such hopes are held out; she sees nothing is to be expected from the part itself; and all that she can do is to make the evil as bearable as possible, by providing against it as well as she can, with what few means she has left.

* There cannot be a doubt (if we admit the hypothesis) that all encysted tumors can only be varieties of the carcinomatous hydatid: since we have daily experience that such occasionally become cancers, therefore such transmutation, together with the marked difference observed in the economy of these tumors in general, and the progress and result of Scirrhi, in common, is left wholly unexplained.

vaginalis testis. By the granulating action of the one, and its quick disposition to heal, you contrast the slow and difficult sloughing of the other; and on this difference you rest a proof of the animalcular existence of these cysts. But here, Sir, the fact only goes to establish a *distinction* between *two* productions; and not by any means a *separate animal economy:* the *distinction* between the living powers of *original* structure, and those of *diseased* formations. No one would ever look for healthy action in a diseased cyst; or, if he did, it is certainly no very great stretch of natural consequences to conclude, that he would be most undoubtedly deceived.* The tedious sloughing of such an exposed cyst can prove nothing for the question. We see the same thing in the slow exfoliations of cartilage: yet, we readily attribute this to a *certain low modification of life,* which such structures possess; and all this without once hinting at the bare possibility of a " separate existence." Alluding, therefore, to the similar proportions of life in the cartilage and the cyst, I cannot conceive, how *that* should establish a *peculiarity* in *disease,* which is freely admitted to be *common* in *health.*†

I beg

---

* No proof of a separate animal economy whatever, is afforded by the comparison of the two cysts. The *active* powers given to a *healthy* or *natural* tunic, attach just as much peculiarity to it, as the *want* of such powers can possibly give to a *diseased* one. It is the *presence* of such powers that constitutes *health*; it is their absence which forms *disease.* So that, in fact, the difference in steatoma to a natural cyst, is that of disease to health; and *not,* what is precisely peculiar to the *separate economy* of the part. If this is not so, and diseased actions, on the contrary, are evidence of a *distinct animal economy*; then, most assuredly, the *same nature* must be allowed in the most unequivocal sense to the tunica vaginalis testis (or any other originally formed cyst), when, from accident, it no longer retains that equilibrium of regenerative powers which constitutes health, and which had afforded, by comparison, that *distinction,* upon which the *separate economy* of encysted tumors (according to the hydatid hypothesis) depends.

† If upon so slender a foundation as that of a *single instance* of similitude between a part and an hydatid, we were immediately to attach to the former, a *distinct* and *peculiar economy*; instead of attending to the *necessities* that are absolutely required to make up a *separate existence,* where would *such* a *mode* of reasoning lead us? We should never have an end of it. In natural as well as moral philosophy, the *first step* is every thing. If animalcular notions got, upon such easy terms, into a mind possessing a strong bias, and creative fancy, how very soon the world might be favoured with " A New System." How very soon he would get from diseased formations to healthy structures; and there, with the aids of *analogy* and *modification,* how very soon the whole would be reduced into *separate* and *peculiar* economies. Full scope in the setting out of the hypothesis is granted,

I beg to trespass a short time longer on your patience, by calling your attention, Sir, to some of the known facts connected with cancer; and the mode by which they are explained in the hydatid hypothesis. There is only one way (if one follows such a mode of reasoning, and which you have adopted) to explain the formation of several schirri in the same breast, at, or about, the same period of time, viz. A disposition to the same production, or growth, in several points at once. Upon any other principle of hydatid growth, this circumstance, in cancer, would be inexplicable; for in the hydatis carcinomatosa, we can have *no bursting of a capsule*, from whence the young hydatids might escape *in a fluid*, as in lymphatica; nor can we admit the other mode of hydatid growth, by *any exterior connection* between these small schirrous bodies and the original or parent schirrus, because the interstices are always filled up by the natural structure of the breast, and therefore with each other they can never be supposed to have borne any relation whatever. So that an hydatid reasoner must content himself in all such instances, by the *bare possibility* of the thing, in contradiction to *probabilities*. But even admitting this point to be explained by your hypothesis, experience presents an insurmountable objection to the hydatid existence of cancer. In the extirpation of schirri in many instances, where these little bodies are present, and have been cut into, they have been found still to present a *natural glandular* appearance, sufficiently so, at any rate, to convince the most firm carcinomatous hypothesist, that such could not be bodies of his class. Now, although such bodies are evidently glands in a certain gradation of change from their original conformations, yet it is not less evident from experience, that they

---

granted, with regard to contents, figuration, and attachments of bodies; so that he would not confine himself, but suit and vary his terms according to the necessities he met. Thus, proceeding on the pure principle of analysis, he would first parcel out the body into hydatis hepatica, hydatis intestinosa, hydatis musculosa, &c. &c. &c. It would be impossible to stop him; the latitude allowed him in the first instance, extends his analogies, and fortifies his conclusions. Having at length given, with some industry and ingenuity, every part its generic name, his synthetical operations would then commence, and the whole would be re-combined under one general economy; and this, with more facility than might, perhaps, be first imagined, aided, as he would be, by the animalcular proof of the ovum, and the analogous growth and clustering of many animalculæ on the same stem. Thus the last touch would be given to this new and stupendous system—The Hydatid Existence of Man!!!

they would progressively have run into a cancerous state; or, in other words, become carcinomatous. So that the only way one can possibly reconcile circumstances to your hypothesis, in this instance, is to conclude (contrary to all experience) that hydatid birth is the *common,* or *necessary* result of glandular alteration.*

Let us now, Sir, compare the circumstances which oftentimes attend the cancerous wart, or excrescence, and the explanation afforded by your hypothesis.—According to your doctrine (if I understand you right), such a wart must have been, from its origin, a carcinomatous hydatid, or a bunch or congeries of the same. When cancer occurs from such an accident as cutting a small excrescence in shaving, it is generally (I believe I may say always) at an advanced age, and the wart till that time has remained, perhaps, twenty or even fifty years, perfectly in a quiescent state. Comparing this quiescent state with hydatid growth, a difficulty arises, but which, in some measure, is accounted for. You tell us, that the torpid state of many schirri is similar to the state of an egg before incubation. This certainly may be admitted in some instances; though the analogy is by no means correct in all; because some schirri will grow rapidly to a *certain size,* and then become *quiescent.* In short, they present so many varieties in their progress, and are evidently so much under the influence of the constitution, participating in many of its sympathies, affected by the slightest causes, irritated at one time, and soothed at another; frequently suffering a partial absorption, and as often wholly taken up; that every circumstance in their natures stands in open contradiction to an animalcular economy.†

But

* Such doctrine would seem most admirably calculated to support the eternity of animal existence; or, in other words, that there would appear to be a provision in the economy of nature, equivalently to make up for the destruction of one existence by the immediate generation of another.

† Schirri, that have been in four years progressive growth, and have taken on a more evident action the last twelve months, have not afforded, after extirpation, the flesh-like appearance, or "fungus," which one would naturally look for, according to the hydatid hypothesis. For we are told, that this "fungus" is invariably produced (from whence *the progress* of cancer) either to separate a dead hydatid from the living; or, when in an advanced state, to select and parcel them out into distinct groups or families, for the purpose of better preservation. But how many schirri are there of long standing, (whose active growth of late obliged their removal)

But to return from this digression to the cancerous excrescence, we will suppose that the quiescent state of a wart, (viewing it as an animalcular existence) say for forty years, and which from some injury has afterwards become cancerous, is explained. But how can we get over the difficulty, according to the hydatid hypothesis, that a cancerous wart shall have been *several times* injured, either by a scratch, bruise, or a cut in shaving, before the fatal accident, which caused it to run into this state; and yet, from *none of* these, did a *similar consequence* follow. For by the hypothesis we are pursuing, (when cancer has followed the shaving off the top of a wart) we must suppose such an injury, either had become a stimulus of growth to the torpid hydatids, or had so injured one or two of them as to occasion their death, from whence sprang up the peculiar "fungus," appropriated for the parting off the *dead* from the *living*. Now, why this should not have happened (since experience shews, that warts have been previously cut with impunity, before they have become cancerous) in the *first* or *second* instance, as well as the *third*, I cannot comprehend, if I am to be a believer in the carcinomatous faith. No one, I believe, will doubt, but what vessels are directly supplied from the surrounding parts, through the substance of the wart. How is this compatible with the existence of an animal independency?

A natural medium is certainly the only way to view disease; however confined its limits may be, it is undoubtedly the only path we have: all the rest is conjectural; and when once we give the rein to fancy, one conjecture is as good as another.

Reflecting thus upon your hypothesis, Sir, one cannot but feel surprised at the extraordinary provision adopted by nature for the preservation of the hydatis carcinomatosa. Here he cannot but see, that, insead of the hypothesis *following*

moval) totally without this fungus. In some, where the absorbents have been actively excited, I have known a line of separation running throughout their whole extent, and a third of an inch in depth. Now, one cannot but feel, if the history of this fungus were correct, that after such a devastation in the very heart of their colony, but what some of the hydatids must have been sufferers, and that some of this fungus would consequently be supplied; this, however, has not taken place. For, after removal, nothing could be seen but a white irregular substance (of cartilaginous compactness throughout) intimately connected with the natural structure of the breast, and having a line of separation running unequally through its surface.

*following nature, she* is made to *follow it.* In pages 44—5—6, and 69, we find her anxiously at work to cherish, prolong, and multiply their existence. To cherish what? To cherish disease! You tell Doctor Baillie, that *his cartilage* in schirrus, before ulceration, is your "primary fungus," generated for the *especial purpose* of fostering the hydatid nurslings; and that when, by ulceration or accident, they become exposed to danger, or to death, a "softer fungus" is *then* produced, which following their growth, carefully cloaks, and selects them into families with all the care of a shepherd, or the anxious solicitude of a mother!

This extraordinary provision for disease, Sir, *alone* would hold me from your faith. I can never be brought to think so ill of Nature. Since the world will have it so, she may be sometimes *whimsical*; her best exertions often fail, and even become diseases; but that she is so base at heart, so palpably false and treacherous to her ends, as your hyypothesis has made her out, I can never admit. All her actions, whatever may be their result, have in the first instance a good intention. When she throws a bag over a stone in the bladder, is it to preserve the stone, or to defend the bladder? Is lamina after lamina of lymph thrown up in aneurism, to prevent the bleeding of the artery, or merely with the intent, at last, from its accumulated bulk, to destroy the surrounding parts? In short, Sir, viewing morbid alterations in general, we find such a uniform attempt *to stop* the progress of disease, instead of *prolonging* it, that such alone is sufficient to exclude the idea of the hydatid existence in cancer; because such an existence can only be supported by imagining a train of operations, disconsonant to general experience, and the known laws of the animal economy.

Even, Sir, your own ability (and which, permit me to say, and not in the way of compliment, is no small proof against your hypothesis) cannot lead you farther with the disease than the breast. After having led one so long through the fertile fields of fancy, your imagination, Sir, is obliged at length to pause. You say, you are at a loss to conjecture for the different appearances of cancer in other parts. Permit me to remark, that, if you had kept in view the original structures of the cancerous breast, and the cancerous lip, their difference in health might have suggested to you a reason for their varieties in disease.

The points of objection to your hypothesis, I submit to be these:

1st.

1st. The solitary fact of the contraction of the cells found in cancerous fat, is not alone sufficient to establish their animalcular existence.

2d. The intimate connection of these cells with the surrounding structure, proves their supply to be in *common* with such parts; and, therefore, such an existence is in direct opposition to an animal independency.

3d. The description and contents of these cells differ so little from, indeed, are so very similar to common adipose, that, so far from their being a *subsequent* production; on the contrary, they would appear to be nothing more than *original* structure, under the change of a morbid progress.

4th. That such an existence is not necessary to the disease, inasmuch as such cellular appearances are not *uniformly* present in cancerous alterations.

5th. The many varieties of the disease; its undefined and irregular progress; the various and dissimilar dispositions of schirri; their frequent participation in the common events of the constitution; the avowed and marked effect of general as well as local means on their nature and structure, appear wholly to exclude the idea of an animalcular economy in cancer.

6th. The admission of such an existence in cancer, renders it necessary to imagine a chain of operations wholly disconsonant to the views of the animal economy, viz. A creation for the sole purpose of preserving and prolonging disease; at the same time that we must entirely exclude, by such an admission, all idea of the known morbid progress, which naturally results from the permanent derangement of parts.

7th. and lastly, Because (although every objection be placed aside, and every position admitted) the hypothesis, after all, is itself imperfect: inasmuch as it is unequal to provide reasons for many of the phenomena attendant on cancer; and when viewed in a collective sense, it is even found inadequate to account for the *common* or *local* varieties of the disease.

These, Sir, are my reasons for rejecting the hydatid existence of cancer, and for the further support of my attempt to explain the origin and progress of this disease, which I have ventured to submit to the public, and which, I know, has been honoured by your perusal.

I am, &c.

SAMUEL YOUNG.

*No. 22, Welbeck Street,*

*March*, 1806.

